# Supplementary material for: Heat adaptation of phage T7 under an extended genetic code
Source: Virus Evol. 2021 Dec 1;7(2):veab100. doi: 10.1093/ve/veab100 (PMC8923235; doi:10.1093/ve/veab100)
Supplement: veab100_Supp [file veab100_supp.zip › Sequencing_Analysis.zip!/Sequencing_Analysis/S2/evidence/JC_77.html]

BRESEQ :: Evidence


| New junction evidence | | | | | | | | | | | |
| --- | --- | --- | --- | --- | --- | --- | --- | --- | --- | --- | --- |
|  | | seq id | position | reads (cov) | reads (cov) | score | skew | freq | annotation | gene | product |
| \* | ? | NC\_001604.1 | = 36882 | 5203 (0.980) | 883 (0.170) | 243/582 | 5.8 | 15.5% | intergenic (–/–) | *–/–* | –/– |
|  | ? | NC\_001604.1 | 37265 = | 4556 (0.890) |  |  |  |  | intergenic (–/–) | *–/–* | –/– |
| Rejected: Coverage evenness skew score above cutoff. | | | | | | | | | | | |
| Rejected: Frequency below/above cutoff threshold. | | | | | | | | | | | |

Only 100 of 1423 total aligned reads displayed.  

`ATTACATTCTTAGAGATGTTGGACACTGCGATGGCTCAGCGTATGCTTGCGGACCTTTCGGACCATGAGCGTCGCTCTCCGCAACTCTATAATGCTATTAACAAACTGTTAGACCGCCACAAGTTCCAGATTGGTAAGTTGCAGCCGGATGTTCACATCTTAGGTGGCCTTGCTGGTGCTCTTGAAGAGTACAAAGAGAAAGTCGGTGATAACGGTCTTACGGATGATGATATTTACACATTACAGTGATATACTCAAGGCCACTACAGATAGTGGTCTTTATGGATGTCATTGTCTATACGAGATGCT‑‑‑‑‑‑‑‑‑‑‑‑‑‑‑‑‑‑‑‑‑‑‑‑‑‑‑‑‑‑‑‑‑‑‑‑‑‑‑‑‑‑‑‑‑‑‑‑‑‑‑‑‑‑‑‑‑‑‑‑‑‑‑‑‑‑‑‑‑‑‑‑‑‑‑‑‑‑‑‑‑‑‑‑‑‑‑‑‑‑‑‑‑‑‑‑‑‑‑‑‑‑‑‑‑‑‑‑‑‑‑‑‑‑‑‑‑‑‑‑‑‑‑‑‑‑‑‑‑‑‑‑‑‑‑‑‑‑‑‑‑‑‑‑‑‑‑‑‑‑‑‑‑‑‑‑‑‑‑‑‑‑‑‑‑‑‑‑‑‑‑‑‑‑‑‑‑‑‑‑‑‑‑‑‑‑‑‑‑‑‑‑‑‑‑‑‑‑‑‑‑‑‑‑‑‑‑‑‑‑‑‑‑‑‑‑‑‑‑‑‑‑‑‑‑‑‑‑‑‑‑‑‑‑‑‑‑‑‑‑‑‑‑‑‑‑‑‑‑‑‑‑‑‑‑‑‑‑‑‑‑‑‑‑‑‑‑‑‑‑‑‑‑‑‑‑‑‑‑‑‑‑‑‑‑‑‑‑‑‑‑‑‑‑‑‑‑‑‑‑  >  NC_001604.1/36574‑36882`  
`‑‑‑‑‑‑‑‑‑‑‑‑‑‑‑‑‑‑‑‑‑‑‑‑‑‑‑‑‑‑‑‑‑‑‑‑‑‑‑‑‑‑‑‑‑‑‑‑‑‑‑‑‑‑‑‑‑‑‑‑‑‑‑‑‑‑‑‑‑‑‑‑‑‑‑‑‑‑‑‑‑‑‑‑‑‑‑‑‑‑‑‑‑‑‑‑‑‑‑‑‑‑‑‑‑‑‑‑‑‑‑‑‑‑‑‑‑‑‑‑‑‑‑‑‑‑‑‑‑‑‑‑‑‑‑‑‑‑‑‑‑‑‑‑‑‑‑‑‑‑‑‑‑‑‑‑‑‑‑‑‑‑‑‑‑‑‑‑‑‑‑‑‑‑‑‑‑‑‑‑‑‑‑‑‑‑‑‑‑‑‑‑‑‑‑‑‑‑‑‑‑‑‑‑‑‑‑‑‑‑‑‑‑‑‑‑‑‑‑‑‑‑‑‑‑‑‑‑‑‑‑‑‑‑‑‑‑‑‑‑‑‑‑‑‑‑‑‑‑‑‑‑‑‑‑‑‑‑‑‑‑‑‑‑‑‑‑‑‑‑‑‑‑‑‑‑‑‑‑‑‑‑‑‑‑‑‑‑‑‑‑‑‑‑‑‑‑‑‑‑cgagatgctAAACGTATTCTCGCAGTGACCCAGAAGGGTGACGCATGGATTCGTGCGTTACAGGATACTATTCGTGAACTGCAACGTAAGTAGGAAATCAAGTAAGGAGGCAATGTGTCTACTCAATCCAATCGTAATGCGCTCGTAGTGGCGCAACTGAAAGGAGACTTCGTGGCGTTCCTATTCGTCTTATGGAAGGCGCTAAACCTACCGGTGCCCACTAAGTGTCAGATTGACATGGCTAAGGTGCTGGCGAATGGAGACAACAAGAAGTTCATCTTACAGGCTTTCCGTGGTATCGGTAAGTCG  >  NC_001604.1/37265‑37564`  

                                                                                                                                                                                                                                                                                                                                                                                                                                                                                                                                                                                                                                   
`ATTACATTCTTAGAGATGTTGGACACTGCGATGGCTCAGCGTATGCTTGCGGACCTTTCGGACCATGAGCGTCGCTCTCCGCAACTCTATACTGCTATTAACAAACTGTTAGACCGCCACAAGTTCCAGATTGGTAAGTTGCAGCCTGATGTTCACATCTTCGGTGGCCTTTCTGTTGCTCTTGAAGCGTACAAAGAGAAAGTCGGTGATAACGGTCTTACGGATGATTATATTTACACATTACAGTGATCTACTCAAGGCCACTACAGCTAGTGGTCTTTATGGATGTCATTGTCTATAC                                                                                                                                                                                                                                                                                                                      >  1:244542/1‑301`  
`ATTACATTCTTAGAGATGTTGGACACTGCGATGGCTCAGCGTATGCTTGCGGACCTTTCGGACCATGAGCGTCGCTCTCCGCAACTCTATAATGCTATTAACAAACTGTTAGACCGCCACAAGTTCCAGATTGGTAAGTTGCAGCCGGATGTTCACATCTTAGGTGGCCTTGCTGGTGCTCTTGAAGAGTACAAAGAGAAAGTCGGTGATAACGGTC‑TACGGATGATGATATTTACACATTACAGTGATATACTCAAGGCCACTACAGATAGTGGTCTTTATGGATGTCATTGTCTATACG                                                                                                                                                                                                                                                                                                                     <  1:448804/301‑1`  
 `gacagTTCTTAGAGATGTTGGACACTGCGATGGCTCAGCGTATGCTTGCGGACCTTTCGGACCATGAGCGTCGCTCTCCGCAACTCTATAATGCTATTAACAAACTGTTAGACCGCCACAAGTTCCAGATTGGTAAGTTGCAGCCGGATGTTCACATCTTAGGTGGCCTTGCTGGTGCTCTTGAAGAGTACAAAGAGAAAGTCGGTGATAACGGTCTTACGGATGATGATATTTACACATTACAGTGATATACTCAAGGCCACTACAGATAGTGGTCTTTATGGATGTCATTGTCTATACG                                                                                                                                                                                                                                                                                                                     <  1:715071/296‑1`  
 `gacagTTCTGAGAGATGTTGGACACTGCGATGGCTCAGCGTATGCTTGCGGACCTTTCGGACCATGAGCGTCGCTCTCCGCAACTCTATAATGCTATTAACAAACTGTTAGACCGCCACAAGTTCCAGATTGGTAAGTTGCAGCCGGATGTTCACATCTTAGGTGGCCTTGCTGGTGCTCTTGAAGAGTACAAAGAGAAAGTCGGTGATAACGGTCTTACGGATGATGATATTTACACATTACAGTGATATACTCAAGGCCACTACAGATAGTGGTCTTTATGGATGTCATTGTCTATACG                                                                                                                                                                                                                                                                                                                     <  1:655354/296‑1`  
 `aacagttaTTAGAGATGTTGTACACTGCGATGGCTCAGCGTATGCTTGCGGACCTTTCGGACCATGAGCGTCGCTCTCCGCAAGTCTATAATGCTATTACCAAACTGTTAGACCGCCACAAGTTCGCGATTGGTAAGTTGCATCCGGATGTTCACATCTTAGGTGGCCTTGCTGGTGCTCTTGAAGAGTACAAAGAGAAAGTCGGTGATAACGGTCTTACGGATGATGATATTTACACATTACAGTGATATACTCAAGGCCACTACAGATAGTGGTCTTTATGGATGTCATTGTCTATACG                                                                                                                                                                                                                                                                                                                     <  1:632968/293‑1`  
 `aacagTTCTTAGAGATGTTGGACCCTGCGATGGCTCAGCGTATGCTTGCGAACCTTTCGGACCATGAGCGTCGCTCTCCGCAACTCTATAATGCTATTAACAAACTGTTAGACCGCCACAAGTTCCAGATTGGTAAGTTGCAGCCGGATGTTCACATCTTAGGTGGCCTTGCTGGTGCTCTTGAAGAGTACAAAGAGAAAGTCGGTGATAACGGTCTTACGGATGATGATATTTACACATTACAGTGATATACTCAAGGCCACTACAGATAGTGGTCTTTATGGATGTCATTGTCTATACG                                                                                                                                                                                                                                                                                                                     <  1:691956/296‑1`  
 `aTACAGTCTTAGAGATGTGGGACACGGCGATGGCTCAGCGTATGCTTGCGGACCTTTCGGAACATGAGCGTCGCTCTACGCAACTCTATAATGCTATTAACAAACTGTTAGACCGCCACAAGTTCCAGATTGGTAAGTTGCAGCCGGATGTTCACATCTTAGGTGGCCTTGCTGGTGCTCTTGAAGAGCACAAAGAGAAAGTCGGTGATAACGGTCTTACGGATGATGATATTTACACATTACAGTGATATACTCAAGGCCACTACAGATAGTGGTCTTTATGGATGTCATTGTCTATACG                                                                                                                                                                                                                                                                                                                     <  1:166976/300‑1`  
 `TTACATTCTTAGAGATGTTGGACACTGCGATGGCTCAGCGTATGCTTGCGGACCTTTCGGACCATGAGCGTCGCTCTCCGCAACTCTATAATGCTATTAACAAACTGTTAGACCGCCACAAGTTCCAGTTTGGTAAGTTGCAGCCGGATGTTCACATCTTAGGTGGCCTTGCTGGTGCTCTTGAAGAGTACAAAGAGAAAGTCGGTGATAACGGTCTTACGGATGATGATATTTACACATTACAGTGATATACTCAAGGCCACTACAGATAGTGGTCTTTATGGATGTCATTGTCTATACG                                                                                                                                                                                                                                                                                                                     <  1:4077/301‑1`  
 `TTACATTCTTAGAGATGTTGGACACTGCGATGGCTCAGCGTATGCTTGCGGACCTTTCGGACCATGAGCGTCGCTCTCCGCAACTCTATAATGCTATTAACAAACTGTTAGACCGCCACAAGTTCCAGATTGGTAAGTTGCAGCCGGATGTTCACATCTTAGGTGGCCTTGCTGGTGCTCTTGAAGAGTACAAAGATAAAGTCGGTGATAACGGTCTTACGGATGATGATATTTACACATTACAGTGATATACTCAAGGCCACTACAGATAGTGGTCTTTATGGATGTCATTGTCTATACG                                                                                                                                                                                                                                                                                                                     <  1:220308/301‑1`  
 `TTACATTCTTAGAGATGTTGGACACTGCGATGGCTCAGCGTATGCTTGCGGACCTTTCGGACCATGAGCGTCGCTCTCCGCAACTCTATAATGCTATTAACAAACTGTTAGACCGCCACAAGTTCCAGATTGGTAAGTTGCAGCCGGATGTTCACATCTTAGGTGGCCTTGCTGGTGCTCTTGAAGAGTACAAAGAGAAAGTCGGTGATAACGGTCTTACGGATGATGATATTTACACATTACAGTGATATACTCAAGGCCACTACAGATAGTGGTCTTTATGGATGTCATTGTCTATACG                                                                                                                                                                                                                                                                                                                     <  1:87576/301‑1`  
 `TTACATTCTTAGAGATGTTGGACACTGCGATGGCTCAGCGTATGCTTGCGGACCTTTCGGACCATGAGCGTCGCTCTCCGCAACTCTATAATGCTATTAACAAACTGTTAGACCGCCACAAGTTCCAGATTGGTAAGTTGCAGCCGGATGTTCACATCTTAGGTGGCCTTGCTGGTGCTCTTGAAGAGTACAAAGAGAAAGTCGGTGATAACGGTCTTACGGATGATGATATTTACACATTACAGTGATATACTCAAGGCCACTACAGATAGTGGTCTTTATGGATGTCATTGTCTATACG                                                                                                                                                                                                                                                                                                                     <  1:558127/301‑1`  
 `TTACATTCTTAGAGATGTTGGACACTGCGATGGCTCAGCGTATGCTTGCGGACCTTTCGGACCATGAGCGTCGCTCTCCGCAACTCTATAATGCTATTAACAAACTGTTAGACCGCCACAAGTTCCAGATTGGTAAGTTGCAGCCGGATGTTCACATCTTAGGTGGCCTTGCTGGTGCTCTTGAAGAGTACAAAGAGAAAGTCGGTGATAACGGTCTTACGGATGATGATATTTACACATTACAGTGATATACTCAAGGCCACTACAGATAGTGGTCTTTATGGATGTCATTGTCTATACG                                                                                                                                                                                                                                                                                                                     <  1:414691/301‑1`  
 `TTACATTCTTAGAGATGTTGGACACTGCGATGGCTCAGCGTATGCTTGCGGACCTTTCGGACCATGAGCGTCGCTCTCCGCAACTCTATAATGCTATTAACAAACTGTTAGACCGCCACAAGTTCCAGATTGGTAAGTTGCAGCCGGATGTTCACATCTTAGGTGGCCTTGCTGGTGCTCTTGAAGAGTACAAAGAGAAAGTCGGTGATAACGGTCTTACGGATGATGATATTTACACATTACAGTGATATACTCAAGGCCACTACAGATAGTGGTCTTTATGGATGTCATTGTCTATACG                                                                                                                                                                                                                                                                                                                     <  1:819681/301‑1`  
 `TTACATTCTTAGAGATGTTGGACACTGCGATGGCTCAGCGTATGCTTGCGGACCTTTCGGACCATGAGCGTCGCTCTCCGCAACTCTATAATGCTATTAACAAACTGTTAGACCGCCACAAGTTCCAGATTGGTAAGTTGCAGCCGGATGTTCACATCTTAGGTGGCCTTGCTGGTGCTCTTGAAGAGTACAAAGAGAAAGTCGGTGATAACGGTCTTACGGATGATGATATTTACACATTACAGTGATATACTAAAGGCCACTACAGATAGTGGTCTTTATGGATGTCATTGTCTATACG                                                                                                                                                                                                                                                                                                                     <  1:627502/301‑1`  
 `TTACATTCTTAGAGATGTTGGACACTGCGATGGCTCAGCGTATGCTTGCGGACCTTTCGGAACATGAGCGTCGCTCTACGCAACTCTATAATGCTATTAACAAACTGTTAGACCGACACAAGTTCCAGATTGGTAAGTTGCAGCCGGATGTTCACATCTTAGGTGGCATTGCTGGTGATCTTGAAGAGTACAAAGAGAAAGTCGGTGATAACGGTCTTACGGATGATGATATTTACACATTACAGTGATATACTCAAGGCCACTACAGATAGTGGTCTTTATGGGTGTCATTGTCTATACG                                                                                                                                                                                                                                                                                                                     <  1:424832/301‑1`  
 `TTACATTCTTAGAGATGTTGGACACTGCGATGGCTCAGCGTATGCTTGCGGACCTTCCGGACCATGAGCGTCGCTCTCCGCAACTCTATAATGCTATTAACAAACTGTTAGACCGCCACAAGTTCCAGATTGGTAAGTTGCAGCCGGATGTTCACATCTTAGGTGGCCTTGCTGGTGATCTTGAAGAGTACAAAGAGAAAGTCGGTGATAACGGTCTTACGGATGATGATATTTACACATTACAGTGATATACTCAAGGCCACTACAGATAGTGGTCTTTATGGATGTCATTGTCTATACG                                                                                                                                                                                                                                                                                                                     <  1:324034/301‑1`  
 `TTACATTCTTAGAGATGTTGGACACTGCGATGGCTCAGCGTATGCGTGCGGACCTTGCGGACCATGAGCGTCGCTCTCCGCAACTCTATAATGCTATTAACAAACTGTTAGACCGCCACAAGTTCCAGATTGGTAAGTTGCAGCCGGATGTTCACATCTTAGGTGGCCTTGCTGGTGCTCTTGAAGAGTACAAAGAGAAAGTCGGTGATAACGGTCTTACGGATGATGATATTTACACATTACAGTGATATACTCAAGGCCACTACAGATAGTGGTCTTTATGGATGTCATTGTCTATACG                                                                                                                                                                                                                                                                                                                     <  1:701709/301‑1`  
 `TTACATGCTTAGAGATGTTGGACACTGCGATGGCTCAGCGTATGCTTGCGGACCTTTCGGACCATGAGCGTCGCTCTCCGCAACTCTATAATGCTATTAACAAACTGTTAGACCGCCACAAGTTCCAGATTGGTAAGTTGCAGCCGGATGTTCACATCTTAGGTGGCCTTGCTGGTGCTCTTGAAGAGTACAAAGAGAAAGTCGGTGATAACGGTCTTACGGATGATGATATTTACACATTACAGTGATATACTCAAGGCCACTACAGATAGTGGTCTTTATGGATGTCATTGTCTATACG                                                                                                                                                                                                                                                                                                                     <  1:455122/301‑1`  
    `CATTCTTAGAGATGTTGGACACTGCGATGGCTCAGCGTATGCTTGCGGACCTTTCGGACCATGAGCGTCGCTCTCCGCAACTCTATAATGCTATTAACAAACTGTTAGACCGCCACAAGTTCCAGATTGGTAAGTTGCAGCCGGATGTTCACATCTTAGGTGGCCTTGCTGGTGCTCTTGAAGAGTACAAAGAGAAAGTCGGTGATAACGGTCTTACGGATGATGATATTTACACATTACAGTGATATACTCAAGGCCACTACAGATAGTGGTCTTTATGGATGTCATTGTCTATACGctg                                                                                                                                                                                                                                                                                                                  <  1:79293/301‑4`  
     `ATTCTTAGAGATGTTGGACACTGCGATGGCTCAGCGTATGCTTGCGGACCTTTCGGACCATGAGCGTCGCTCTCCGCAACTCTATAATGCTATTAACAAACTGTTAGACCGCCACAAGTTCCAGATTGGTAAGTTGCAGCCGGATGTTCACATCTTAGGTGGCCTTGCTGGTGCTCTTGAAGAGTACAAAGAGAAAGTCGGTGATAACGGTCTTACGGATGATGATATTTACACATTACAGTGATATACTCAAGGCCACTACAGATAGTGGTCTTTATGGATGTCATTGTCTATACGAGAT                                                                                                                                                                                                                                                                                                                 <  1:172060/301‑1`  
     `ATTCTTAGAGATGTTGGACACTGCGATGGCTCAGCGTATGCTTGCGGACCTTTCGGACCATGAGCGTCGCTCTCCGAAACTCTATAATGCTATTAACAAACTGTTAGACAGCCACAAGTTCCAGATTGGTAAGTTGCAGCCGGATGGTCACATCGTAGGTGGCCTTGCTGGTGCTCTTGAAGAGTACAAAGAGAAAGTCGGTGATAACGGTCTTACGGATGATGATATTTACACATTACAGTGATATACTCAAGGCCACTACAGATAGTGGTCTTTATGGATGTCATTGTCTATACGAGAT                                                                                                                                                                                                                                                                                                                 <  1:100049/301‑1`  
     `ATTCTTAGAGATGTTGGACACTGCGATGGCTCAGCGTATGCTTGCGGACCTTTCGGACCATGAGCGTCGATCTCCGCAACTCTATAATGCTATTAACAAACTGTTATACCGCCACAAGTGCCAGATTGGTAAGTTGCAGCCGGATGTTCACATCTTAGGTGGCCTTGCTGGTGCTCTTGAAGAGTACAAAGAGAAAGTCGGTGATAACGGTCTTACGGATGATGATATTTACACATTACAGTGATATACTCAAGGCCACTACAGATAGTGGTCTTTATGGATGTCATTGTCTATACGAGAT                                                                                                                                                                                                                                                                                                                 <  1:177664/301‑1`  
      `TTCTTAGAGATGTTGGACACTGCGATGGCTCAGCGTATGCTTGCGGACCTTTCGGACCATGAGCGTCGCTCTCCGCAACTCTATAATGCTATTAACAAACTGTTAGACCGCCACAAGTTCCAGATTGGTAAGTTGCAGCCGGATGTTCACATCTTAGGTGGCCTTGCTGGTGCTCTTGAAGAGTACAAAGAGAAAGTCGGTGATAACGGTCTTACGGATGATGATATTTACACATTACAGTGATATACTCAAGGCCACTACAGATAGTGGTCTTTATGGATGTTATTGTCTATACGAGAct                                                                                                                                                                                                                                                                                                                >  1:372122/1‑299`  
      `TTCTTAGAGATGTTGGACACTGCGATGGCTCAGCGTATGCTTGCGGACCTTTCGGACCATGAGCGTCGCTCTCCGCAACTCTATAATGCTATTAACAAACTGTTAGACCGCCACAAGTTCCAGATTGGTAAGTTGCAGCCGGATGTTCACATCTTAGGTGGCCTTGCTGGTGCTCTTGAAGAGTACAAAGAGAAAGTCGGTGATAACGGTCTTACGGATGATGATATTTACACATTACAGTGATATACTCAAGGCCACTACAGATAGTGGTCTTTATGGATGTCATTGTCTATACtctttc                                                                                                                                                                                                                                                                                                                >  1:540972/1‑295`  
      `TTCTTAGAGATGTTGGACACTGCGATGGCTCAGCGTATGCTTGCGGACCTTTCGGACCATGAGCGTCGCTCTCCGCAACTCTATAATGCTATTAACAAACTGTTAGACCGCCACAAGTTCCAGATTGGTAAGTTGCAGCCGGATGTTCACATCTTAGGTGGCCTTGCTGGTGCTCTTGAAGAGTACAAAGAGAAAGTCGGTGATAACGGTCTTACGGATGATGATATTTACACATTACAGTGATATACTCAAGGCCACTACAGATAGTGGTCTTTATGGATGTCATTGTCTATACGAGATG                                                                                                                                                                                                                                                                                                                >  1:384960/1‑301`  
      `TTCTTAGAGATGTTGGACACTGCGATGGCTCAGCGTATGCTTGCGGACCTTTCGGACCATGAGCGTCGCTCTCCGCAACTCTATAATGCTATTAACAAACTGTTAGACCGCCACAAGTTCCAGATTGGTAAGTTGCAGCCGGATGTTCACATCTTAGGTGGCCTTGCTGGTGCTCTTGAAGAGTACAAAGAGAAAGTCGGTGATAACGGCCTTACGGATGATGATATTTACACATTACAGTGATATACTCAAGGCCACTACAGATAGTGGTCTTTATGGATGTCATTGTCTATACGAGCTG                                                                                                                                                                                                                                                                                                                >  1:798821/1‑301`  
       `TCTTAGAGATGTTGGACACTGCGATGGCTCAGCGTATGCTTGCGGACCTTTCGGACCATGAGCGTCGCTCTCCGCAACTCTATAATGCTATTAACAAACTGTTAGACCGCCACAAGTTCCAGATTGGTAAGTTGCAGCTGGATGTTCACATCTTAGGTGGCCTTGCTGGTGCTCTTGAAGAGTACAAAGAGAAAGTCGGTGATAACGGTCTTACGGATGATGATATTTACACATTACAGTGATATACTCAAGGCCACTACAGATAGTGGTCTTTATGGATGTCATTGTCTATACGctgtct                                                                                                                                                                                                                                                                                                               >  1:204743/1‑295`  
        `CTTAGAGATGTTGGACACTGCGATGGCTCAGCGTATGCTTGCGGACCTTTCGGACCATGAGCGTCGCTCTCCGCAACTCTATAATGCTATTAACAAACTGTTAGACCGACACAAGTTCCAGATTGGTAAGTTGCAGCCGGATGTTCACATCTTAGGTGGCCTTGCTGGTGCTCTTGAAGAGTACAAAGAGAAAGTCGGTGATAACGGTCTTACGGATGATGATATTTACACATTACAGTGATATACTCAAGGCCACTACAGATAGTGGTCTTTATGGATGTCATTGTCTATACGAGATGCT                                                                                                                                                                                                                                                                                                              >  1:578184/1‑301`  
               `ATGTTGGACACTGCGATGGCTCAGCGTATGCTTGCGGACCTTTCGGACCATGAGCGTCGCTCTCCGCAACTCTATAATGCTATTAACAAACTGTTAGACCGCCACAAGTTCCAGATTGGTAAGTTGCAGCCGGCTGTTCACATCTTAGGTGGCCTTGCTGGTGCTCTTGAATAGTACAAATAGAAAGTCGGTGATAACGGTCTTACGGATGCTGATATTTACACATTACAGTGATATACTCAATGCCTCTACAGCTAGTGGTCTTTATGGATGTCATTGTCTATACTAGATGCTAAACGTt                                                                                                                                                                                                                                                                                                       >  1:339655/1‑300`  
                 `GTTGGACACTGCGCTGGCTCAGCGTATGCTTGCGGACCTTTCGGACCATGAGCGTCGCTCTCCGCAACTCTATAATGCTATTAACAAACTGTTAGACCGCCACAAGTTCAAGATTGGTAAGTTGCAGCCGGATGTTCACATCTTAGGTGGCCTTGCTGGTGCTCTTGAAGAGTACCAAGAGAAAGTCGGTGATAACGGTCTTACGGATGATGATATTTACAATTTCCAGTGATATACTCAAGGCCACTACAGATAGTGGTCTTTATGGCTGTCATTGTCTATACGAGATGCTAAACGTATT                                                                                                                                                                                                                                                                                                     >  1:160463/1‑301`  
                         `CTGCGATGGCTCAGCGTATGCTTGCGGACCTTTCGGACCATGAGCGTCGCTCTCCGCAACTCTATAATGCTATTAACAAACTGTTAGACCGCCACAAGTTCCAGATTGGTAAGTTGCAGCCGGATGTTCACATCTTAGGTGGCCTTGCTGGTGCTCTTGAAGAGTACAAAGAGAAAGTCGGTGATAACGGTCTTACGGATGATGATATTTACACATTACAGTGATATACTCAAGGCCACTACAGATAGTGGACTTTCTGGATGTCATTGTCTATCCGAGATGCTAAACGTATTCTCGCAGT                                                                                                                                                                                                                                                                                             >  1:549548/1‑301`  
                                      `GCGTATGCTTGCGGACCTTTCGGACCATGAGCGTCGCTCTCCGCAACTCTATAATGCTATTAACAAACTGTTAGACCGCCACAAGTTCCAGATTGGTAAGTTGCAGCCGGATGTTCACATCTTAGGTGGCCTTGCTGGTGCACTTGAAGAGGACAAAGAGAAAGTCGGTGATAACGGTCTTACGGATGATGATATTTACACATTACAGTGATATACTCAAGGCACCTACAGATAGTGGTATTTTTGGATGTCATTGTCTATACGAGATGCTAAAGGTATTCTCGCAGGTACTCAAAAGGtt                                                                                                                                                                                                                                                                                >  1:494145/1‑299`  
                                            `GCTTGCGGACCTTTCGGACCATGAGCGTCGCTCTCCGCAACTCTATAATGCTATTAACAAACTGTTAGACCGCCACAAGTTCCAGATTGGTAAGTTGCAGCCGGATGTTCACATCTTAGGTGGCCTTGCTGGTGCTCTTGAAGAGTACAAAGAGAAAGTCGGTGATAACGGTCTTACGGATGATGATATTTACACATTACAGTGATATACTCAAGGCCACTACATATAGTGGTCTTTATGGATGTCCTTGTCTATACGAGATGCTAAACGTATTCTCGCAGTGACCCAGAAGGGTGACGCg                                                                                                                                                                                                                                                                          >  1:660681/1‑300`  
                                                                   `tgtgtataagagacagACTCTATAATGCTATTAACAAACTGTTAGACCGCCACAAGTTCCAGATTGGTAAGTTGCAGCCGGATGTTCACATCTTAGGTGGCCTTGCTGGTGCTCTTGAAGAGTACAAAGAGAAAGTCGGTGATAACGGTCTTACGGACGATGATATTTACACATTACAGTGATATACTCAAGGCCACTACAGATAGTGGTCTTTATGGATGTCATTGTCTATACGAGATGCTAAACGTATTCTCGCAGTGACCCAGAAGGGTGACGCATGGATTCGTGCGTTACAGGATAC                                                                                                                                                                                                                                                   <  1:534127/285‑1`  
                                                                   `gGCGTCGCTCTCCGCAACTCTATAATGCTATTAACAAACTGTTAGACCGCCACAAGTTCCAGATTGGTAAGTTGCAGCCGGATGTTCACATCTTAGGTGGCCTTGCTGGTGCTCTTGAAGAGTACAAAGAGAAAGTCGGTGATAACGGTCTTACGGATGATGATATTTACACATTACAGTGATATACTCAAGGCCACTACAGATAGTGGTCTTTATGGATGTCATTGTCAATACGAGATGCTAAACGTATTCTCGCAGTGACCCAGAAGGGTGACGCATGGATTCGTGCGTTACAGGATAC                                                                                                                                                                                                                                                   <  1:525831/300‑1`  
                                                                      `tgtgtataagagacagCTATAATGCTATTAACAAACTGTTAGACCGCCACAAGTTCCAGATTGGTAAGTTGCAGCCGGATGTTCACATCTTAGGTGGCCTTGCTGGTGCTCTTTAAGAGTACAAAGAGAAAGTCGGTGATAACGGTCTTACGGATGATGATATTTACACATTACAGTGATAAACTCAAGGCCACTACAGATAGTGGTCTTTATGGATGTCATTGTCTATACGAGATGCTAAACGTATTCTCGCAGTGACCCAGAAGGGTGACGCATGGATTCGTGCGTTACAGGATACTAT                                                                                                                                                                                                                                                <  1:285613/285‑1`  
                                                                              `CCGCAACTCTATAATGCTATTAACAAACTGTTAGCCCGCCACAAGTTCCAGATTGGTAAGTTGCAGCCGGATGTTCACATCTTAGGTGGCCTTGCTGGTGCTCTTTAAGAGTACACAGAGAAAGTCGGTGATAACGTTCTTACGGATGATGATATTTACACATTACAGTGATATACTCAAGGCCACTACAGATAATGGTCTTTATGGATGTCATTGTCTATACGATATGCTAAACGTATTCTCGCAGTTACCCAGAAGGGTGACGCATGGATTCGTGAGGTACAGGATACTATTAGTGAAa                                                                                                                                                                                                                                        >  1:61706/1‑300`  
                                                                                   `ACTCTATAATGCTATTAACAAACTGTTAGACCGCCACAAGTTCCTGATTGGTAAGTTGCATCCGGATGTTCACATCTTAGGTGGCCTTGCTGGTGCTCTTGAAGAGTACAAAGAGAAAGTCGGTGATAACGGTCTTACGGATGATGATATTTACACATTACAGTGATATACTCAAGGCCACTACAGATAGTGGTCTTTATGGATGTCATTGTCTATACGAGATGCTAAACGTATTCTCGCAGTGACCCAGAAGGGTGACGCATGGATTCGTGCGTTACAGGATACctgtctcttatacaca                                                                                                                                                                                                                                   >  1:281353/1‑285`  
                                                                                   `ACTCTATAATGCTATTAACAAACTGTTAGACCGCCACAAGTTCCAGATTGGTAAGTTGCAGCCGGATGTTCACATCTTAGGTGGCCTTGCTGGTGCTCTTGAAGAGTACAAAGAGAAAGTAGGTGATAACGGTCTTACGGCTGATGATATTTACACATTACAGTGATATACTCAAGGCCACTACAGATAGTGGTCTTTATGGATGTCATTGTCTATACGAGATGCTAAACGTATTCTCGCAGTGACCCAGAAGGGTGACGCATGGATTCGTGCGTTACAGGATACTATTCGTGAACTGCAA                                                                                                                                                                                                                                   >  1:112970/1‑301`  
                                                                                        `ATAATGCTATTAACAAACTGTTAGACCGCCACAAGTTCCAGATTGGTAAGTTGCAGCCGGATGTTCACATCTTAGGTGGCCTTGCTGGTGCTCTTGAAGAGTACAAAGAGAAAGTCGGTGATAACGGTCTTACGGATGATGATATTTACACATTACAGTGATATACTCAAGGCCACTACAGATAGTGGTCTTTATGGATGTCATTGTCTATACGAGATGCTAAACGTATTCTCGCAGTGACCCAGAAGGGTGACGCATGGATTCGTGCGTTACAGGATACTATTCGTGctgtctcttatac                                                                                                                                                                                                                              >  1:573211/1‑288`  
                                                                                        `ATAATGCTATTAACAAACTGTTAGACCGCCACAAGTTCCAGATTGGTAAGTTGCAGCCGGATGTTCACATCTTAGGTGGCCTTGCTGGTGCTCTTGAAGAGTACAAAGAGAAAGTCGGTGATAACGGTCTTACGGATGATGATATTTACACATTACAGTGATATACTCAAGGCCACTACAGATAGTGGTCTTTATGGATGTCATTGTCTATACGAGATGCTAAACGTATTCTCGCAGTGACCCAGAAGGGTGACGCATGGATTCGTGCGTTACAGGATACTATTCGTGAACTGCAACGTAA                                                                                                                                                                                                                              <  1:378670/301‑1`  
                                                                                             `GCTATTAACAAACTGTTAGACCGCCACAAGTTCCAGATTGGTAAGTTGCAGCCGGATGTTCACATCTTAGGTGGCCTTGCTGGTGCTCTTGAAGAGTACAAAGAGAAAGTCGGTGATAACGGTCTTACGGATGATGATATTTACACATTACAGTGATATACTCAAGGCCACTACAGATAGTGGTCTTTATGGATGTCATTGTCTATACGAGATGCTAAACGTATTCTCGCAGTGACCCAGAAGGGTGACGCATGGATTCGTGCGTTACAGGATACTATTCGTGTACTGCAACGTctgtctc                                                                                                                                                                                                                         >  1:107532/1‑294`  
                                                                                                `ATTAACAAACTGTTAGACCGCCACAAGTTCCAGATTTGTAAGTTGCAGCCGGATGTTCACATCTTAGGTGGCCTTGCTGGTGCTCTTGAAGAGTACAAAGAGAAAGTCGGTGATAACGGTCTTACGGATGATGATATTTACACATTACAGTGATATACTCAAGGCCACTACAGATAGTGGTCTTTATGGATGTCATTGTCTATACGAGATGCTAAACGTATTCTCGCAGTGACCCAGAAGGGTGACGCATGGATTCGTGCGTTACAGGATACTATTCGctgtctcttatacacatctccga                                                                                                                                                                                                                      >  1:139306/1‑278`  
                                                                                                         `CTGTTAGACCGCCACAAGTTCCAGATTGGTAAGTTGCAGCCGGATGTTCACATCTTAGGTGGCCTTGCTGGTGCTCTTGAAGAGTACAAAGAGAAAGTCGGTGATAACGGTCTTACGGATGATGATATTTACACATTACAGTGATATACTCAAGGCCACTACAGATAGTGGTCTTTATGGATGTCATTGTCTATACGAGATGCTAAACGTATTCTCGCAGTGACCCAGAAGGGTGACGCATGGATTCGTGCGTTACAGGATACTATTCGTGAACTGCAACGTAAGTAGGAAATCAAGTAAG                                                                                                                                                                                                             >  1:190299/1‑301`  
                                                                                                         `CTGTTAGACCGCCACAAGTTCCAGATTGGTAAGTTGCAGCCGGATGTTCACATCTTAGGTGGCCTTGCTGGTGCTCTTGAAGAGTACAAAGAGAAAGTCGGTGATAACGGTCTTACGGATGATGATATTTACACATTACAGTGATATACTCAAGGCCACTACAGACAGTGGTCTTTATGGATGTCATTGTCTATACGAGATGCTAAACGTATTCTCGCAGTGACCCAGAAGGGTGACGCATGGATTCGTGCGTTACAGGATACTATTCGTGAACTGCAACGTACGCAGGAAATCAAGTAAG                                                                                                                                                                                                             >  1:708863/1‑301`  
                                                                                                              `AGACCGCCACAAGTTCCAGATTGGTAAGTTGCAGCCTGATGTTCACATCTTAGGTGGCCTTGCTGGTGCTCTTGAAGAGTACAAAGAGAAAGTCGGTGATAACGGTCTTACGGATGATGATATTTACACATTACAGTGATATACTCAAGGCCACTACAGATAGTGGTCTTTATGGATGTCATTGTCTATACGAGATGCTAAACGTATTCTCGCAGTGACCCAGAAGGGTGACGCATGGATTCGTGCGTTACAGGATACTATTCGTGAACTGCAACGTAAGTAGGAAATCAAGTAAGGAGGC                                                                                                                                                                                                        <  1:213592/301‑1`  
                                                                                                                                   `gtcggagatgtgtataagagacagCATCTTAGGTGGCCTTGCTGGTGCTCTTGAAGAGTACAAAGAGAAAGTCGGTGATAACGGTCTTACGGATGATGATATTTACACATTACAGTGATATACTCAAGGCCACTACAGATAGTGGTCTTTATGGATGTCATTGTCTATACGAGATGCTAAACGTATTCTCGCAGTGACCCAGAAGGGTGACGCATGGATTCGTGCGTTACAGGATACTATTCGTGAACTGCAACGTAAGTAGGAAATCAAGTAAGGAGGCAATGTGTCTACTCAATCCAAT                                                                                                                                                                                   <  1:363685/277‑1`  
                                                                                                                                                                       `CCTTGCTGGTTCTCTTGAAGAGTAAAAAGAGAAAGTCGGTGATAACGGTCTTACGGATGATGATATGTACACATTACAGTGATATACTCAAGGCCACTACAGATAGTGGTCTTTATGGATGTCATTGTCTATACGAGATGCTAAACGTATTCTCGCAGTGACCCAGAAGGGTGACGCATGGATTCGTGCGTTACAGGATACTATTCGTGAACTGCAACGTAAGTAGGAAATCAAGTAAGGAGGCAATGTGTCTACTCAATCCAATCGTAATGCGCTCGTAGTGGCGCAACTGAAAGGAGAC                                                                                                                                               <  1:749291/301‑1`  
                                                                                                                                                                          `TGCTGGTGCTCTTGAAGAGTACAAAGAGAAAGTCGGTGATAACGGTCTTACGGATGATGATATTTACACATTACAGTGATATACTCAAGGCCACTACAGATAGTGGTCTTTATGGATGTCATTGTCTATACGAGATGCTAAACGTATTCTCGCAGTGACCCAGAAGGGTGACGCATGGATTCGTGCGTTACAGGATACTATTCGTGAACTGCAACGTAAGTAGGAAATCAAGTAAGGAGGCAATGTGTCTACTCAATCCAATCGTAATGCGCTCGTAGTGGCGCAACTGAAAGGAGACTTC                                                                                                                                            >  1:260423/1‑301`  
                                                                                                                                                                                 `GCTCTTGAAGAGTACAAAGAGAAAGTCGGTGATAACGGTCTTACGGATGATGATATTTACACATTACAGTGATATACTCAAGGCCACTACAGATAGTGGTCTTTATGGATGTCATTGTCTATACGAGATGCTAAACGTATTCTCGCAGTGACCCAGACGGGTGACGCATGGATTCGTGCGTTACAGGATACTATTCGTGAACTGCAACGTAAGTAGGAAATCAAGTAAGGAGGCAATGTGTCTACTCAATCCAATCGTAATGCGCTCGTAGTGGAGCAACTGAAAGGAGACTTCTTGGCGT                                                                                                                                     >  1:740076/1‑301`  
                                                                                                                                                                                  `CTCTTGAAGAGTACAAAGAGAAAGTCGGTGATAACGGTCTTACGGATGATGATATTTACACATTACAGTGATATACTCAAGGCCACTACAGATAGTGGTCTTTATGGATGTCATTGTCTATACGAGATGCTAAACGTATTCTCGCAGTGACCCAGAAGGGTGACGCATGGATTCGTGCGTTACAGGATACTATTCGTGAGCTGCAACGTAAGTAGGAAATCAAGTAAGGAGGCAATGTGTCTACTCAATCCAATCGTAATGCGCTCGTAGTGGCGCAACTGAAAGGAGACTTCGTGGCctg                                                                                                                                    >  1:155312/1‑298`  
                                                                                                                                                                                       `GAAGAGTACAAAGAGAAAGTCGGTGATAACGGTCTTACGGATGATGATATTTACACATTACAGTGATATACTCAAGGCAACTACAGATAGTGGTCTTTATGGATGTCATTGTCTATACGAGATGCTAAACGTCTTCTCGCTGTGACCCAGAAGGGTGACGCATGGATTCGTACGTTACAGGATACTATTCGTGAACTGCAACGTAAGTAGGAAATCAAGTAAGGAGGCAATGTGTATACTCAATCCAATCGTAATGCGCTCGTAGTGGCGCAACTGAAATGAGCCTTCtgtctcttatacc                                                                                                                               >  1:230065/1‑288`  
                                                                                                                                                                                        `aaaagagacagGAGAAAGTCGGTGATAACGGTCTTACTGATAATGATATTTACACATTACAGTGAAATACTAAAGGCCACTACAGATAGTGGTCTTTATGGATGTCATTGTCTATACGAGATGCTAAACGTATTCTCGCAGTGACCCAGAAGGGTGACGCATGGATTCGTGCGTTACAGGATACTATTCGTGAACTGCAACGTAAGTAGGAAATCAAGTAAGGAGGCAATGTGTCTACTCAATCCAATCGTAATGCGCTCGTAGTGGCGCAACTGAAAGGAGACTTCGTGGCGTTCCTATT                                                                                                                              <  1:588948/290‑1`  
                                                                                                                                                                                            `GTACAAAGAGAAAGTCGGTGATAACGGTCTTACGGATGATGATATTTACACATTACAGTGATATACTCAAGGCCACTACAGATAGTGGTCTTTATGGATGTCATTGTCTATACGAGATGCTAAACGTATTCTCGCAGTGACCCAGAAGGGTGACGCATGGATTCGTGCGTTACAGGATACTATTCGTGAACTGCAACGTAAGTAGGAAATCAAGTAAGGAGGCAATGTGTCTACTCAATCCAATCGTAATGCGCTCGTAGTGGCGCAACTGAAAGGAGACTTCGctgtctcttatacacat                                                                                                                          >  1:593844/1‑284`  
                                                                                                                                                                                                              `gtgggctcggagatgtgtataagagacagACACATTACAGTGATATACTCAAGGCCACTACAGATAGTGGTCTTTATGGATGTCATTGTCTATACGAGATGCTAAACGTATTCTCGCAGTGACCCAGAAGGGTGACGCATGGATTCGTGCGTTACAGGATACTATTCGTGAACTGCAACGTAAGTAGGAAATCAAGTAAGGAGGCAATGTGTCTACTCAATCCAATCGTAATGAGCTCGTAGTGGCGCAACTGAAAGGAGACTTCGTGGCGTTCCTATTCGTCTTATGGAAGGCGCTAAAC                                                                                                        <  1:780069/272‑1`  
                                                                                                                                                                                                                     `GGTCTTACGGATGATGATATTTACACATTACAGTGATATACTCAAGGCCACTACAGATAGTGGTCTTTATGGATGTCATTGTCTATACGAGATGCTAAACGTATTCTCGCAGTGACCCAGAAGGGTGACGCATGGATTCGTGCGTTACAGGATACTATTCGTGAACTGCAACGTAAGTAGGAAATCAAGTAAGGAGGCAATGTGTCTACTCAATCCAATCGTAATGCGCTCGTAGTGGCGCAACTGAAAGGAGACTTCGTGGCGTTCCTATTCGTCTTATGGAAGGCGCTAAACCTACCGG                                                                                                 <  1:487179/301‑1`  
                                                                                                                                                                                                                      `GTCTTACGGATGATGATATTTACACATTACAGTGATATACTCAAGGCCACTACAGATAGTGGTCTTTATGGATGTCATTGTCTATACGAGATGCTAAACGTATTCTCGCAGTGACCCAGAAGGGTGACGCATGGATTCGTGCGTTACAGGTTACTATTCGTGAACTGCAACGTAAGTAGGAAATCAAGTAAGGAGGCAATGTGTCTACTCAATCCAATCGTAATGCGCTCGTAGTGGCGCAACTGAAAGGAGACTTCGTGGCGTTCCTATTCGTCTgtctcttatacacatctccgagccc                                                                                                >  1:18320/1‑276`  
                                                                                                                                                                                                                        `tgtataagagacagTATTTACACATTACAGTGATATACTCAAGGCCACAACAGATAGTGGTCTTTATGGATGTCATTGTCTATACGAGATGCTAAACGTATTCTCGCAGTGACCCAGAAGGGTGACGCATGGATTCGTGCGTTACAGGATACTATTCGTGAACTGCAACGTAAGCAGGAAATCAAGTAAGGAGGCAATGTGTCTACTCAATCCAATCGTAATGCGCTCGTAGTGGCGCAACTGAAAGGAGACTTCGTGGCGTTCCTATTCGTCTTATGGAAGGCGCTAAACCTACCGGTGC                                                                                              <  1:509476/287‑1`  
                                                                                                                                                                                                                          `TACGGATGATGATATTTACACATTACAGTGATATACTCAAGGCCACTACAGATAGTGGTCTTTATGGATGTCATTGTCTATACGAGATGCTAAACGTATTCTCGCAGTGACCCAGAAGGGTGACGCATGGATTCGTGCGTTACAGGATACTATTCGTGAACTGCAACGTAAGTAGGAAATCAAGTAAGGAGGCAATGTGTCTACTCAATCCAATCGTAATGCGCTCGTAGTGGCGCAACTGAAAGGAGACTTCGTGGCGTTCCTATTCGTCTTATGGAAGGCGCTAAACCTACCGGTGCCC                                                                                            <  1:132850/301‑1`  
                                                                                                                                                                                                                            `aGGATGATGAGATTTACACATTACAGTGATATACTCAAGGCCACTACAGATAGTGGTCTTTATGGATGTCATTGTCTATACGAGATGCTAAACGTATTCTCGCAGTGACCCAGAAGGGTGACGCATGGATTCGTGCGTTACAGGATACTATTCGTGAACTGCAACGTAAGTAGGAAATCAAGTAAGGAGGCAATGTTTCTACTCAATCCAATCGTAATGCGCTCGTAGTGGCGCAACTGAAAGGAGACTTCGTGGCGTTCCTATTCGTCTTATGGAAGGCGCTAAACCTACCGGTGCCCAC                                                                                          <  1:42937/300‑1`  
                                                                                                                                                                                                                                    `gagacagACACATTACAGTGATATACTCAAGGCCACTACAGATAGTGGTCTTTATGGATGTCATTGTCTATACGAGATGCTAAACGTATTCTCGCAGTGACCCAGAAGGGTGACGCATGGATTCGTGCGTTACAGGATACTATTCGTGAACTGCAACGTAAGTAGGAAATCAAGTAAGGAGGCAATGTGTCTACTCAATCCAATCGTAATGCGCTCGTAGTGGCGCAACTGAAAGGAGACTTCGTGGCGTTCCTATTCGTCTTATGGAAGGCGCTAAACCTACCGGTGCCCACTAAGTGTC                                                                                  <  1:641730/294‑1`  
                                                                                                                                                                                                                                      `TATTTACACATTACAGTGATATACTCAAGGCCACTACAGATAGTGGTCTTTATGGATGTCATTGTCTATACGAGATGCTAAACGTATTCTCGCAGTGACCCAGAAGGGTGACGCATGGATTCGTGCGTTACAGGATACTATTCGTGAACTGCAACGTAAGTAGGAAATCAAGTAAGGAGGCAATGTGTCTACTCAATCCAATCGTAATGCGCTCGTAGTGGCGCAACTGAAAGGAGACTTCGTGGCGTTCCTATTCGTCTTATGGAAGGCGCTAAACCTACCGGTGCCCACTAAGTGTCAG                                                                                <  1:147404/301‑1`  
                                                                                                                                                                                                                                             `ACATTACAGTGATATACTCAAGGCCACTACAGATAGTGGTCTTTATGGATGTCATTGTCTATACGAGATGCTAAACGTATTCTCGCAGTGACCCAGAAGGGTGACGCATGGATTCGTGCGTTACAGGATACTATTCGTGAACTGCAACGTAAGTAGGAAATCAAGTAAGGAGGCAATGTGTCTACTCAATCCAATCGTAATGCGCTCGTAGTGGCGCAACTGAAAGGAGACTTCGTGGCGTTCCTATTCGTCTTATGGAAGGCGCTAAACCTACCGGTGCCCACTAAGTGTCAGATTGACA                                                                         >  1:403258/1‑301`  
                                                                                                                                                                                                                                                `TTACAGTGATATACTCAAGGCCACTACAGATAGTGGTCTTTATGGATGTCATTGTCTATACGAGATGCTAAACGTATTCTCGCAGTGACCCAGAAGGGTGACGCATGGATTCGTGCGTTACAGGATACTATTCGTGAACTGCAACGTAAGTAGGAAATCAAGTAAGGAGGCAATGTGTCTACTCAATCCAATCGTAATGCGCTCGTAGTGGCGCAACTGAAAGGAGACTTCGTGGCGTTCCTATTCGTCTTATGGAAGGCGCTAAACCTACCGGTGCCCACTAAGTGTCAGATTGACATGG                                                                      >  1:768167/1‑301`  
                                                                                                                                                                                                                                                `TTACAGTGATATACTCAAGGCCACTACAGATAGTGGTCTTTATGGATGTCATTGTCTATACGAGATGCTAAACGTATTCTCGCAGTGACCCAGAAGGGTGACGCATGGATTCGTGCGTTACAGGATACTATTCGTGAACTGCAACGTAAGTAGGAAATCAAGTAAGGAGGCAATGTGTCTACTCAATCCAATCGTAATGCGCTCGTAGTGGCGCAACTGAAAGGAGACATCGTGGCGTTCCTATTAGTCTTCTGGAAGGCGCTAAACCTACCGGTGCCCACTAAGTGTCAGATTGACATGG                                                                      >  1:94394/1‑301`  
                                                                                                                                                                                                                                                      `gGATATACTCAAGGCCACTACAGATAGTGGTCTTTATGGATGTCATTGTCTATACGAGATGCTAAACGTATTCTCGCAGTGACCCAGAAGGGTGACGCATGGATTCGTGCGTTACAGGATACTATTCGTGAACTGCAACGTAAGTAGGAAATCAAGTAAGGAGGCAATGTGTCTACTCAATCCAATCGTAATGCGCTCGTAGTGGCGCAACTGAAAGGAGACTTCGTGGCGTTCCTATTCGTCTTATGGAAGGCGCTAAACCTACCGGTGCCCACTAAGTGTCAGATTGACATGGCTAAGG                                                                <  1:564165/300‑1`  
                                                                                                                                                                                                                                                      `atgtgtataagagacagCTACAGATAGTGGTCTTTATGGATGTCATTGTCTATACGAGATGCTAAACGTATTCTCGCAGTGACCCAGAAGGGTGACGCATGGATTCGTGCGTTACAGGATACTATTCGTGAACTGCAACGTAAGTAGGAAGTCAAGTAAGGAGGCAATGTGTCTACTCAATCCAATCGTAATGCGCTCGTAGTGGCGCAACTGAAAGGAGACTTCGTGGCGTTCCTATTCGTCTTATGGAAGGCGCTAAACCTACCGGTGCCCACTAAGTGTCAGATTGACATGGCTAAGG                                                                <  1:316371/284‑1`  
                                                                                                                                                                                                                                                      `agaggtgtataagagacagACAGATAGTGGGCTTTATGGATGTCATTGTCTATACGAGATGCTAAACGTATTCTCGCAGTGACCCAGAAGGGTGACGCATGGATTCGTGCGTTACAGGATACTATTCGTGAACTGCAACGTAAGTAGGAAATCAAGTAAGGAGGCAATGTGTCTACTCAATCCAATCGTAATGCGCTCGTAGTGGCGCAACTGAAAGGAGACTTCGTGGCGTTCCTATTCGTCTTATGGAAGGCGCTAAACCTACCGGTGCCCACTAAGTGTCAGATTGACATGGCTAAGG                                                                <  1:306422/282‑1`  
                                                                                                                                                                                                                                                      `TGATATACTCAAGGCCACTACAGATAGTGGTCTTTATGGATGTCATTGTCTATACGAGATGCTAAACGTATTCTCGCAGTGACCCAGAAGGGTGACGCATGGATTCGTGCGTTACAGGATACTATTCGTGAACTGCAACGTAAGTAGGAAATCAAGTAAGGAGGCAATGTGTCTACTCAATCCAATCGTAATGCGCTCGTAGTGGCGCAACTGAAATGAGACTTCGTGGCGTTCCTATTCGTCTTATGGAAGGCGCTAAACCTACCGGTGCCCACTAAGTGTCAGATTGACATGGCTAAGG                                                                <  1:12526/301‑1`  
                                                                                                                                                                                                                                                       `GATATACTCAAGGCCACTACAGATAGTGGTCTTTATGGATGTCATTGTCTATACGAGATGCTAAACGTATTCTCGCAGTGACCCAGAAGGGTGACGCATGGATTCGTGCGTTACAGGATACTATTCGTGAACTGCAACGTAAGTAGGAAATCAAGTAATGAGGCAATGTGTCTACTCAATCCAATCGTAATGCGCTCGTAGTGGCGCAACTGAAAGGAGACTTCGTGGCGTTCCTATTCGTCTTATGGAAGGCGCTAAACCTACCGGTGCCCACTAAGTGTCAGATTGACATGGCTAAGGg                                                               >  1:479720/1‑300`  
                                                                                                                                                                                                                                                       `GATATACTCAAGGCCACTACAGATAGTGGTCTTTATGGATGTCATTGTCTATACGAGATGCTAAACGTATTCTCGCAGTGACCCAGAAGGGTGACGCATGGATTCGTGCGTTACAGGATACTATTCGTGAACTGCAACGTAAGTAGGAAATCAAGTAAGGAGGCAATGTGTCTACTCAATCCAATCGTAATGCGCTCGTAGTGGCGCAACTGAAAGGAGACTTCGTGGCGTTCCTATTCGTCTTATGTAAGGAGCTAAACCTACCGGTGCCCACTAAGTGTCAGATTGACATGGCTACGGT                                                               >  1:121422/1‑301`  
                                                                                                                                                                                                                                                       `GATATACTCAAGGCCACTACAGATAGTGGTCTTTATGGATGTCATTGTCTATACGAGATGCTAAACGTATTCTCGCAGTGACCCAGAAGGGTGACGCATGGATTCGTGCGTTACAGGATACTATTCGTGAACTGCAACGTAAGTAGGAAATCAAGTAAGGAGGCAATGTGTCTACTCAATCCAATCGTAATGCGCTCGTAGTGGCGCAACTGAAAGGAGACTTCGTGGCGTTCCTATTCGTCTTATGGAAGGCGCTAAACCTACCGGTGCCCACTAAGTGTCAGATTGACATGGCTctgtc                                                               >  1:734303/1‑296`  
                                                                                                                                                                                                                                                          `ATACTCAAGGCCACTACAGATAGTGGTCTTTATGGATGTCATTGTCTATACGAGATGCTAAACGTATTCTCGCAGTGACCCAGAAGGGTGACGCATGGATTCGTGCGTTACAGGATACTATTCGTGAACTGCAACGTAAGTAGGAAATCAAGTAAGGAGGCAATGTGTCTACTCAATCCAATCGTAATGCGCTCGTAGTGGCGCAACTGAAAGGAGACTTCGTGGCGTTCCTATTCGTCTTATGGAAGGAGCTAAACCTACCGGTGCCCACTAAGTGTCAGATctgtctcttatacacatc                                                            >  1:517494/1‑283`  
                                                                                                                                                                                                                                                            `ACTCAAGGCCACTACAGATAGTGGTCTTTATGGATGTCATTGTCTATACGAGATGCTAAACGTATTCTCGCAGTGACCCAGAAGGGTGACGCATGGATTCGTGCGTTACAGGATACTATTCGTGAACTGCAACGTAAGCAGGAAATCAAGTAAGGAGGCAATGTGTCTACTCAATCCAATCGTAATGCGCTCGTAGTGGCGCAACTGAAAGGAGACTTCGTGGCGTTCCTATTCGTCTTATGGAAGGCGCTAAACCTACCGGTGCCCACTAAGTGTCAGATTGACATGGCTAAGGTGCTGG                                                          >  1:436913/1‑301`  
                                                                                                                                                                                                                                                              `TCAAGGCCACTACAGATAGTGGTCTTTATGGATGTCATTGTCTATACGAGATGCTAAACGTATTCTCGCAGTGACCCAGAAGGGTGACGCATGGATTCGTGCGTTACAGGATACTATTCGTGAACTGCAACGTAAGTAGGAAATCAAGTAAGGAGGCAATGTGTCTACTCAATCCAATCGTAATGCGCTCGTAGTGGCGCAACTGAAAGGAGACTTCGTGGCGTTCCTATTCGTCTTATGGAAGGCGCTAAACCTACCGGTGCCCACTAAGTGTCAGATTGGCATGGCTAAGGTGCTGGCG                                                        <  1:499873/301‑1`  
                                                                                                                                                                                                                                                              `TCAAGGCCACTACAGATAGTGGTCTTTATGGATGTCATTGTCTATACGAGATGCTAAACGTATTCTCGCAGTGACCCAGAAGGGTGACGCATGGATTCGTGCGTTACAGGATACTATTCGTGAACTGCAACGTAAGTAGGAAATCAAGTAAGGAGGCAATGTGTCTACTCAATCCAATCGTAATGCGCTCGTAGTGGCGCAACTGAAAGGAGACTTCGTGGCGTTCCTATTCGTCTTATGGAAGGCGCTAAACCTACCGGTGCCCACTAAGTGTCAGATTGACATGGCTAAGGTGCTGGCG                                                        <  1:350335/301‑1`  
                                                                                                                                                                                                                                                                       `CTACAGATAGTGGTCTTTATGGATGTCATTGTCTATACGAGATGCTAAACGTATTCTCGCAGTGACCCAGAAGGGTGACGCATGGATTCGTGCGTTACAGTATACTATTCGTGAACTGCAACGTAAGTAGGAAATCAAGTACGGAGGCAATGTGTCTACTCAATCCAATCGTAATGCGCTCGTAGTGGCGCAACTGACAGGAGACTTCGTGGCGATCCTATTCGTCTTTTGGAAGCCGCTAATCCTACCGGTGCCTACTCAGTGTCAGTTTGACATGGCTAAGGTGCTGGCGAATGGAGAC                                               >  1:471671/1‑301`  
                                                                                                                                                                                                                                                                       `CTACAGATAGTGGTCTTTATGGATGTCATTGTCTATACGAGATGCTAAACGTATTCTCGCAGTGACCCAGAAGGGTGACGCATGGATTCGTGCGTTACAGGATACTATTCGTGAACTGCAACGTAAGTAGGAAATCAAGTAAGGAGGCAATGTGTCTACTCAATCCACTCGTAATGCGCTCGTAGTGGCGCAACTGAAAGGAGACTTCGTGGCGTTCCTATTCGTCTTATGGAAGGCGCTAAACCTACCGGTGCCCACTAAGTGACAGATTGACATGGCTAAGGTGCTGGCGAATGGAGAC                                               >  1:394087/1‑301`  
                                                                                                                                                                                                                                                                       `CTACAGATAGTGGTCTTTATGGATGTCATTGTCTATACGAGATGCTAAACGTATTCTCGCAGTGACCCAGAAGGGTGACGCATGGATTCGTGCGTTACAGGATACTATTCGTGAACTGCAACGTAAGTAGGAAATCAAGTAAGGAGGCAATGTGTCTACTCAATCCAATCGTAATGCGCTCGTAGTGGCGCAACTGAAAGGAGACTTCGTGGCGTTCCTATTCGTCTTATGGAAGGCGCTAAACCTACCGGTGCCCACTAAGTGTCAGATTGACATGGCTAAGGTGCTGGCGAATGGAGAC                                               >  1:268777/1‑301`  
                                                                                                                                                                                                                                                                           `AGATAGTGGTCTTTATGGATGTCATTGTCTATACGAGATGCTAAACGTATTCTCGCAGTGACACAGAAGGGTGACGCATGGATTCGTGCGTTACAGGATACTATTCGTGAACTGCAACGTAAGTAGGAAATCAAGTAAGGAGGCAATGTGTCTACTCAATCCAATCGCAATGCGCTCGTAGTGGCGCAACTGAAAGGAGACTTCGTGGCGTTCCTATTCGTCTTATGGAAGGCGCTAAACCTACCGGTGCCCACTAAGTGTCAGATTGACATGGCTAAGGTGCTGGCGAATGGAGACAACA                                           <  1:252356/301‑1`  
                                                                                                                                                                                                                                                                            `GATAGTGGTCTTTATGGATGTCATTGTCTATACGAGATGCTAAACGTATTCTCGCAGTGACCCAGAAGGGTGACGCATGGATTCGTGCGTTACAGGATACTATTCGTGAACTGCAACGTAAGCAGGAAATCAAGTAAGGAGGCAATGTGTCTACTCAATCCAATCGTAATGCGCTCGTAGTGGCGCAACTGAAAGGAGACTTCGTGGCGTTCCTATTCGTCTTATGGAAGGCGCTAAACCTACCGGTGCCCACTAAGTGTCAGATTGACATGGCTAAGGTGCTGGCTAATGGAGACAACAA                                          >  1:299585/1‑301`  
                                                                                                                                                                                                                                                                                   `gatgtgtataagagacagGTCTATACGAGATGCTAAACGTATTCTCGCAGTGACCCAGAAGGGTGACGCATGGATTCGTGCGTTACAGGATACTATTCGTGAACTGCAACGTAAGTAGGAAATCAAGTAAGGAGGCAATGTGTCTACTCAATCCAATCGTAATGCGCTCGTAGTGGCGCAACTGAAAGGAGACTTCGTGGCGTTCCTATTTGTCTTATGGAAGGCGCTAAACCTACCGGTGCCCACTAAGTGTCAGATTGACATGGCTAAGGTGCTGGCGAATGGAGACAACAAGAAGTTC                                   <  1:671051/283‑1`  
                                                                                                                                                                                                                                                                                   `GTCTTTATGGATGTCATTGTCTATACGAGATGCTAAACGTATTCTCGCAGTGACCCAGAAGGGTTACGCATGGATTCGTGCGTTACAGGATACTATTCGTGAACTGCAACGTAAGTAGGAAATCAAGTAAGGAGGCAATGTGTCTACTCAATCCAATCGTAATGCGCTCGTAGTGGCGCAACTGAAAGGAGACTTCGTGGCGTTCCTATTCGTCTTATGGACGGCGCTAAACCTACCGGTGCTCACTAAGTGTCTGATTGACATGGCTCAGGTGCTGGCGAATGTCGACAACAAGAAGTTt                                   >  1:276287/1‑300`  
                                                                                                                                                                                                                                                                                    `TCTTTATGGATGTCATTGTCTATACGAGATGCTAAACGTATTCTCGCAGTGACCCAGAAGGGTGACGCATGGATTCGTGCGTTACAGGATACTATTCGTGAACTGCAACGTAAGTAGGAAATCAAGTAAGGAGGCAATGTGTCTACTCAATCCAATCGTAATGCGCTCGTAGTGGCGCAACTGAAAGGAGACTTCGTGGCGTTCCTATTCGTCTTATGGAAGGCGCTAAACCTACCGGTGCCCACTAAGTGTCAGATTGACATGGCTAAGGTGCTGGCGACTGGAGACAACAAGAAGTTCA                                  >  1:724064/1‑301`  
                                                                                                                                                                                                                                                                                              `gGTCATTTTCTATACGAGATGCTAAACGTATTCTCGAAGTGACCCAGAAGGGTGACGGATGGATTCGTGCGTTACAGGATACTATTCGTGAACTGCAACGTAAGTAGGAAATCAAGTAAGGAGGCAATGTGTCTACTCAATCCAATCGTAATGCGCTCGTAGTGGCGCAACTGAAAGGAGACTTCGTGGCGTTCCTATTCGTCTTATGGAAGGCGCTAAACCTACCGGTGCCCACTAAGTGTCAGATTGACATGGCTAAGGTGCTGGCGAATGGAGACAACAAGAAGTTCATCTTACAGGC                        <  1:198191/300‑1`  
                                                                                                                                                                                                                                                                                              `atgtgtataagagacagGATGCTAAACGTATTTCCGCAGTGACCCAGAAGGGTGACGCATGGATTCGTGCGTTACAGGATACTATTCGTGAACTGCAACGTAAGCAGGAAATCAAGTAAGGAGGCAATGTGTCTACTCAATCCAATCGTAATGCGCTCGTAGTGGCGCAACTGAAAGGAGACTTCGTGGCGTTCCTATTCGTCTTATGGAAGGCGCTAAACCTACCGGTGCCCACTAAGTGTCAGATTGACATGGCTAAGGTGCTGGCGAATGGAGACAACAAGAAGTTCATCTTACAGGC                        <  1:827789/284‑1`  
                                                                                                                                                                                                                                                                                                  `tgtgtataagagacagGCTAAACGTATTCTCGCAGTGACCCAGAAGGGTGACGCATGGATTCGTGCGTTACAGGAGACTATTCGTGAACTGCAACGTAAGCAGGAAATCAAGTAAGGAGGCAATGTGTCTACTCAATCCAATCGTAATGCGCTCGTAGTGGCGCAACTGAAAGGAGACTTCGTGGCGTTCCTATTCGTCTTATGGAAGGCGCTAAACCTACCGGTGCCCACTAAGTGTCAGATTGACATGGCTAAGGTGCTGGCGAATGGAGACAACAAGAAGTTCATCTTACAGGCTTTC                    <  1:23779/285‑1`  
                                                                                                                                                                                                                                                                                                     `GTCTATACGAGATGCTAAACGTATTCTCGCAGTGACCCAGAAGGGTGACGCATGGATTCGTGCGTTCCAGGATACTATTCGTGAACTGCAACGTAAGTAGGAAATCAAGTAAGGAGGCAATGTGTCTACTCCATCCAATCGTAATGCGCTCGTAGTGGCGCAACTGAAAGGAGACTTCGTGGCGTTCCTATTCGTCTTATGGAAGGCGCTACACCTACCGGTGCCCACTAAGTGTCAGATTGACATGGCTAAGGTGCTGGCGAATGGAGACAACAAGAAGTTCATCTTACAGGCTTTCCGT                 >  1:46378/1‑301`  
                                                                                                                                                                                                                                                                                                              `AGATGCTAAACGTATTCTCGCAGTGACCCAGAAGGGTGACGCATGGATTCGTGCGTTACAGGATACTATTCGTGAACTGCAACGTAAGTAGGAAATCAAGTAAGGAGGCAATGTGTCTACTCAATCCAATCGTAATGCGCTCGTAGTGGCGCAACTGAAAGGAGACTTCGTGGCGTTCCTATTCGTCTTATGGAAGGCGCTAAACCTACCGGTGCCCACTAAGTGTCAGATTGACATGGCTAAGGTGCTGGCGAATGGAGACAACAAGAAGTTCATCTTACAGGCTTTCCGTGGTATCGGT        >  1:356311/1‑301`  
                                                                                                                                                                                                                                                                                                              `AGATGCTAAACGTATTCTCGCAGTGACCCAGAAGGGTGACGCATGGATTCGTGCGTTACAGGATACTATTCGTGAACTGCAACGTAAGCAGGAAATCAAGTAAGGAGGCAATGTGTCTACTCAATCCAATCGTAATGCGCTCGTAGTGGCGCAACTGAAAGGAGACTTCGTGGCGTTCCTATTCGTCTTATGGAAGGCGCTAAACCTACCGGTGCCCACTAAGTGTCAGATTGACATGGCTAAGGTGCTGGCGAATGGAGACAACAAGAAGTTCATCTTACAGGCTTTCCGTGGTATCGGT        <  1:290085/301‑1`  
                                                                                                                                                                                                                                                                                                               `agTGCTAAACGTATTCTCGCAGTGACCCAGAAGGGTGACGCATGGATTCGTGCGTTACAGGATACTATTCGTGAACTGCAACGTAAGTAGGAAATCAAGTAAGGAGGCAATGTGTCTACTCAATCCAATCGTAATGCGCTCGTAGTGGCGCAACTGAAAGGAGACTTCGTGGCGTTCCTATTCGTCTTATGGAAGGCGCTAAACCTACCGGTGCCCACTAAGTGTCAGATTGACATGGCTAAGGTGCTGGCGAATGGAGACAACAAGAAGTTCATCTTACAGGCTTTCCGTGGTATCGGTA       <  1:757807/299‑1`  
                                                                                                                                                                                                                                                                                                               `GATGCTAAACGTATTCTCGCAGTGACCCAGAAGGGTGACGCATGGATTCGTGCGTTACAGGATACTATTCGTGAACTGCAACGTAAGTAGGAAATCAAGTAAGGAGGCAATGTGTCTACTCAATCCAATCGTAATGCGCTCGTAGTGGCGCAACTGAAAGGAGACTTCGTGGCGTTCCTATTCGTCTTATGGAAGGCGCTAAACCTACCGGTGCCCACTAAGTGTCAGATTGACATGGCTAAGGTGCTGGAGAATGGAGACAACAAGAAGTTCtgtctcttatacacatctccgagcccac       >  1:810219/1‑273`  
                                                                                                                                                                                                                                                                                                               `GATGCTAAACGTATTCTCGCAGTGACCCAGAAGGGTGACGCATGGATTCGTGCGTTACAGGATACTATTCGTGAACTGCAACGTAAGTAGGAAATCAAGTAAGGAGGCAATGTGTCTACTCAATCCAATCGTAATGCGATCGTAGTGGCGCAACTGAAAGGAGACTTCGTGGCGTTCCTATTCGTCTTATGGAAGGCGCTAAACCTACCGGTGCCCACTAAGTGTCAGATTGACATGGCTAAGGTGCTGGCGAATGGAGACAACAAGAAGTTCGTCTTACAGGCTTTCCGTGGTATCGGTA       >  1:606708/1‑301`  
                                                                                                                                                                                                                                                                                                               `GATGCTAAACGTATTCTCGCAGTGACCCAGAAGGGTGACGCATGGATTCGTGCGTTACAGGATACTATTCGTGAACTGCAACGTAAGCAGGAAATCAAGTAAGGAGGCAATGTGTCTACTCAATCCAATCGTAATGCACTCGTAGTGGCGCAACTGAAAGGAGACTTCGTGGCGTTCCTATTCGTCTTATGGAAGGCGCTAAACCTACCGGTGCCCACTAAGTGTCAGATTGACATGGCTAAGGTGCTGGCGAATGGAGACAACAAGAAGTTCATCTTACAGGCTTTCCGTGGTATCGGTA       >  1:333435/1‑301`  
                                                                                                                                                                                                                                                                                                                `ATGCTAAACGTATTCTCGCAGTGACCCAGAAGGGTGACGCATGGATTCGTGCGTTACAGGATACTATTCGTGAACTGCAACGTAAGCAGGAAATCAAGTAAGGAGGCAATGTGTCTACTCAATCCAATCGTAATGCGCTCGTAGTGGCGCAACTGAAAGGAGACTTCGTGGCGTTCCTATTCGTCTTATGGAAGGCGCTAAACCTACCGGTGCCCACTAAGTGTCCGATTGACATGGCTAAGGTGCTGGCGAATGGAGACAACAAGAAGTTCATCTTACAGGCTTTCCGTGGTATCGGTAA      >  1:680566/1‑301`  
                                                                                                                                                                                                                                                                                                                 `TGCTAAACGTATTCTCGCAGTGACCCAGAAGGGTGACGCATGGATTCGTGCGTTACAGGATACTATTCGTGAACTGCAACGTAAGTAGGAAATCAAGTAAGGAGGCAATGTGTCTACTCAATCCAATCGTAATGCGCTCGTAGTGGCGCAACTGAAAGGAGACTTCGTGGCGTTCCTATTCGTCTTATGGAAGGCGCTAAACCTACCGGTGCCCACTAAGTGTCAGATTGACATGGCTAAGGTGCTGGCGAATGGAGACAACAAGAAGTTCATCTTACAGGCTTTCCGTGGTATCGGTAAG     <  1:696169/301‑1`  
                                                                                                                                                                                                                                                                                                                 `TGCTAAACGTATTCTCGCAGTGACCCAGAAGGGTGACGCATGGATTCGTGCGTTACAGGATACTATTCGTGAACTGCAACGTAAGCAGGAAATCAAGTAAGGAGGCAATGTGTCTACTCAATCCAATCGTAATGCGCTCGTAGTGGCGCAACTGAAAGGAGACTTCGTGGCGTTCCTATTCGTCTTATGGAAGGCGCTAAACCTACCGGTGCCCACTAAGTGTCAGATTGACATGGCTAAGGTGCTGGCGAATGGAGACAACAAGAAGTTCATCTTACAGGCTTTCCGTGGTATCGGTAAG     <  1:685760/301‑1`  
                                                                                                                                                                                                                                                                                                                  `GCTAAACGTATTCTCGCAGTGACCCAGAAGGGTGACGCATGGATTCGTGCGTTACAGGATACTATTCGTGAACTGCAACGTAAGTAGGAAATCAAGTAAGGAGGCAATGTGTCTACTCAATCCAATCGTAATGCGCTCGTAGTGGCGCAACCGAAAGGAGACTTCGTGGCGTTCCTATTCGTCTTATGGAAGGCGCTAAACCTACCGGTGCCCACTAAGTGTCAGATTGACATGGCTAAGGTGCTGGCGAATGGAGACAACAAGAAGTTCATCTTACAGGCTTTCCGTGGTATCGGTAAGT    >  1:647707/1‑301`  
                                                                                                                                                                                                                                                                                                                  `GCTAAACGTATTCTCGCAGTGACCCAGAAGGGTGACGCATGGATTCGTGCGTTACAGGATACTATTCGTGAACTGCAACGTAAGCAGGAAATCAAGTAAGGAGGCAATGTGTCTACTCAATCCAATCGTAATGCGCTCGTAGTGGCGCAACTGAAAGGAGACTTCGTGGCGTTCCTATTCGTCTTATGGAAGGCGCTAAACCTACCGGTGCCCACTACGTGTCAGATTGACATGGCTAAGGTGCTGGCGAATGGAGACAACAAGAAGTTCATCTTACAGGCTTTCCGTGGTATCGGTAAGT    >  1:599144/1‑301`  
                                                                                                                                                                                                                                                                                                                  `GCTAAACGTATTCTCGCAGTGACCCAGAAGGGTGACGCATGGATTCGTGCGTTACAGGATACTATTCGTGAACTGCAACGTAAGCAGGAAATCAAGTAAGGAGGCAATGTGTCTACTCAATCCAATCGTAATGCGCTCGTAGTGGCGCAACTGAAAGGAGACTTCGTGGCGTTCCTATTCGTCTTATGGAAGGCGCTAAACCTACCGGTGCCCACTAAGTGTCAGATTGACATGGCTAAGGTGCTGGCGAATGGAGACAACAAGAAGTTCATCTTACAGGCTTTCCGTGGTATCTGTctct    >  1:623224/1‑297`  

                                                                                                                                                                                                                                                                                                                                                                                                                                                                                                                                                                                                                                   
`ATTACATTCTTAGAGATGTTGGACACTGCGATGGCTCAGCGTATGCTTGCGGACCTTTCGGACCATGAGCGTCGCTCTCCGCAACTCTATAATGCTATTAACAAACTGTTAGACCGCCACAAGTTCCAGATTGGTAAGTTGCAGCCGGATGTTCACATCTTAGGTGGCCTTGCTGGTGCTCTTGAAGAGTACAAAGAGAAAGTCGGTGATAACGGTCTTACGGATGATGATATTTACACATTACAGTGATATACTCAAGGCCACTACAGATAGTGGTCTTTATGGATGTCATTGTCTATACGAGATGCT‑‑‑‑‑‑‑‑‑‑‑‑‑‑‑‑‑‑‑‑‑‑‑‑‑‑‑‑‑‑‑‑‑‑‑‑‑‑‑‑‑‑‑‑‑‑‑‑‑‑‑‑‑‑‑‑‑‑‑‑‑‑‑‑‑‑‑‑‑‑‑‑‑‑‑‑‑‑‑‑‑‑‑‑‑‑‑‑‑‑‑‑‑‑‑‑‑‑‑‑‑‑‑‑‑‑‑‑‑‑‑‑‑‑‑‑‑‑‑‑‑‑‑‑‑‑‑‑‑‑‑‑‑‑‑‑‑‑‑‑‑‑‑‑‑‑‑‑‑‑‑‑‑‑‑‑‑‑‑‑‑‑‑‑‑‑‑‑‑‑‑‑‑‑‑‑‑‑‑‑‑‑‑‑‑‑‑‑‑‑‑‑‑‑‑‑‑‑‑‑‑‑‑‑‑‑‑‑‑‑‑‑‑‑‑‑‑‑‑‑‑‑‑‑‑‑‑‑‑‑‑‑‑‑‑‑‑‑‑‑‑‑‑‑‑‑‑‑‑‑‑‑‑‑‑‑‑‑‑‑‑‑‑‑‑‑‑‑‑‑‑‑‑‑‑‑‑‑‑‑‑‑‑‑‑‑‑‑‑‑‑‑‑‑‑‑‑‑‑‑  >  NC_001604.1/36574‑36882`  
`‑‑‑‑‑‑‑‑‑‑‑‑‑‑‑‑‑‑‑‑‑‑‑‑‑‑‑‑‑‑‑‑‑‑‑‑‑‑‑‑‑‑‑‑‑‑‑‑‑‑‑‑‑‑‑‑‑‑‑‑‑‑‑‑‑‑‑‑‑‑‑‑‑‑‑‑‑‑‑‑‑‑‑‑‑‑‑‑‑‑‑‑‑‑‑‑‑‑‑‑‑‑‑‑‑‑‑‑‑‑‑‑‑‑‑‑‑‑‑‑‑‑‑‑‑‑‑‑‑‑‑‑‑‑‑‑‑‑‑‑‑‑‑‑‑‑‑‑‑‑‑‑‑‑‑‑‑‑‑‑‑‑‑‑‑‑‑‑‑‑‑‑‑‑‑‑‑‑‑‑‑‑‑‑‑‑‑‑‑‑‑‑‑‑‑‑‑‑‑‑‑‑‑‑‑‑‑‑‑‑‑‑‑‑‑‑‑‑‑‑‑‑‑‑‑‑‑‑‑‑‑‑‑‑‑‑‑‑‑‑‑‑‑‑‑‑‑‑‑‑‑‑‑‑‑‑‑‑‑‑‑‑‑‑‑‑‑‑‑‑‑‑‑‑‑‑‑‑‑‑‑‑‑‑‑‑‑‑‑‑‑‑‑‑‑‑‑‑‑‑cgagatgctAAACGTATTCTCGCAGTGACCCAGAAGGGTGACGCATGGATTCGTGCGTTACAGGATACTATTCGTGAACTGCAACGTAAGTAGGAAATCAAGTAAGGAGGCAATGTGTCTACTCAATCCAATCGTAATGCGCTCGTAGTGGCGCAACTGAAAGGAGACTTCGTGGCGTTCCTATTCGTCTTATGGAAGGCGCTAAACCTACCGGTGCCCACTAAGTGTCAGATTGACATGGCTAAGGTGCTGGCGAATGGAGACAACAAGAAGTTCATCTTACAGGCTTTCCGTGGTATCGGTAAGTCG  >  NC_001604.1/37265‑37564`

  

Alignment Legend

Aligned base mismatch/match (shaded by quality score): `ATCG/ATCG` `< 3 ≤` `ATCG/ATCG` `< 13 ≤` `ATCG/ATCG` `< 27 ≤` `ATCG/ATCG` `< 36 ≤` `ATCG/ATCG` `< 38 ≤` `ATCG/ATCG`

Unaligned base: `atcg`    Masked matching base: `atcg`    Alignment gap: `‑`    Deleted base: `‑`

Reads not counted as support for junction

read\_name Not counted due to insufficient overlap past the breakpoint.

read\_name Not counted due to not crossing MOB target site duplication.
